# Post-stroke Depression Increases Disability More Than 15% in Ischemic Stroke Survivors: A Case-Control Study

**DOI:** 10.3389/fneur.2019.00926

**Published:** 2019-08-27

**Authors:** Stefano Paolucci, Marco Iosa, Paola Coiro, Vincenzo Venturiero, Anna Savo, Domenico De Angelis, Giovanni Morone

**Affiliations:** Fondazione Santa Lucia-IRCCS, Rome, Italy

**Keywords:** stroke, depression, rehabilitation, antidepressants, functional outcome

## Abstract

We performed a retrospective, case-control study in consecutive ischemic stroke patients admitted to our stroke rehabilitation unit. Patients were matched for severity of neurological impairment (evaluated with the Canadian Neurological Scale, CNS), age (difference within 1 year), and onset admission interval (difference within 3 days). Participants were divided into two subgroups according to the presence or absence of PSD. Aim was to assess the specific influence of post-stroke depression (PSD) and antidepressant treatment on both basal functional status and rehabilitation outcomes. All PSD patients were treated primarily with serotoninergic antidepressants (AD). The final sample included 280 patients with depression (out of 320 found in a whole case series of 993 ischemic patients, i.e., 32.25%) and 280 without depression. Forty patients with depression were excluded because they had a history of severe psychiatric illness or aphasia, with a severe comprehension deficit. On one hand, PSD patients obtained lower Barthel Index (BI) and Rivermead Mobility Index (RMI) scores at both admission and discharge, with minor effectiveness of rehabilitative treatment and longer length of stay; on the other hand, this group had a lower percentage of dropouts. Lastly, PSD patients showed a different functional outcome, based on their response to antidepressant therapy, that was significantly better in responders than in non-responders (13.13%). Our results confirm the unfavorable influence of PSD on functional outcome, despite pharmacological treatment.

## Introduction

Post-stroke depression (PSD) is one of the most frequent neuropsychiatric consequences of stroke. It affects almost 30% of stroke survivors (1), and thus may greatly influence the prognosis, not only “quoad valetudinem” (of health) but also “quoad vitam” (of life). In fact, patients with PSD have an increased risk of mortality (have an increased risk of mortality, both short- and long-term (2–4), a higher risk of suicidality (5), increased cognitive impairment (6, 7), increased risk of falls (8), increased hospitalization costs (9), and a poorer quality of life (10–12).

Moreover, PSD has a negative impact on functional outcome and rehabilitation results. In fact, PSD is associated with increased disability (13–15), reduced participation in rehabilitative programs (16, 17), and worse rehabilitation results (18–22). Conversely, a reduction of depressive symptoms has been associated with better functional recovery (23); furthermore, patients treated for depression show a better functional prognosis compared to untreated, depressed patients (24, 25). Clearly, PSD is only one of the potential risk factors used to evaluate stroke functional outcome. Stroke severity, increasing age and timing of rehabilitation are determining factors for disability after stroke (26–30). Moreover, the multivariate models used in most outcome studies tend to be specific but less sensitive and they do not allow for a careful evaluation of the specific role of each factor in functional outcome. Therefore, to understand the specific role of each potential prognostic factor in functional outcome, it is necessary to avoid or reduce the role of other well-recognized risk factors.

The aim of this study was to evaluate the specific weight of both basal functional status and rehabilitation outcomes in PSD in consecutive ischemic stroke inpatients after matching for neurological severity, age and onset-admission interval (OAI) to rule out the influence of these most powerful prognostic factors. In particular, the study compared basal functional status and rehabilitation outcomes in homogeneous subgroups of stroke patients who were admitted for rehabilitation of first stroke sequelae. They were homogenous for severity of neurological impairment (evaluated with the Canadian Neurological Scale, CNS), age (difference within 1 year) and onset admission interval (difference within 3 days). They were divided into two subgroups according to the presence or absence of PSD.

## Methods

### Subject Selection

From a whole case series of 993 consecutive ischemic stroke patients admitted to our rehabilitation unit between January 2004 and December 2018 for sequelae of a first event, we retrospectively found 320 cases with a clinical diagnosis of PSD (32.25%). We excluded 40 patients with depression, 6 patients (1.87%) who had a history of severe psychiatric illness and 34 (10.62%) who had aphasia with a severe comprehension deficit. Therefore, the final sample included 280 patients with depression. We used only data collected retrospectively from patients' medical records. The data were anonymized so that individuals could not be identified. Because of the study design, there were no risks, disadvantages or infringements to individual and/or family rights. Upon admission, a formal informed consent form, which allowed for the analysis of clinical data for the purposes of this study, was signed by all patients.

The exclusion criteria for this study were the following: previous cerebrovascular accidents, hemorrhagic stroke, subarachnoid hemorrhage, presence of language disorder with severe comprehension deficits (see methods), previous severe depression before stroke (with history of admission to psychiatric ward), presence of other chronic disabling pathologies (i.e., severe Parkinson's disease, polyneuropathy, severe cardiac, liver or renal failure, cancer, and limb amputation).

Our rehabilitation ward is located in a large, free-standing, university-affiliated rehabilitation institute that is not part of a general acute hospital. Following the request of the latter, admission is allowed for all recent stroke survivors who have severe or moderate functional disability or cognitive loss (ICD-9 diagnosis code 438) but no severe medical conditions that contraindicate physical therapy. The threshold criteria for hospital admission are the ability to actively participate in rehabilitation and tolerate intense daily treatment.

Stroke has been defined as a sudden, non-convulsive, focal neurological deficit that persists for more than 24 h (31). In this study, the diagnosis of stroke was based on personal history, clinical examination and neuroradiological findings (i.e., all patients underwent computed tomography [CT] scans or magnetic resonance imaging [MRI]).

### Clinical, Neurological, and Functional Assessment

Upon admission, all patients were submitted to clinical, neurological, neuropsychological, neuroradiological, and functional examinations. The rehabilitation staff included physicians (physiatrists, neurologists, urologists, and otorhinolaryngologists), neuropsychologists, nurses, physiotherapists, occupational and speech therapists, a social services care manager, dietitians, and support staff.

We used the revised and validated version of the CNS to measure stroke severity, with a cut-off score of 11.5 for normal patients (32).

Functional data included rehabilitation length of stay, number and characteristics of dropouts, activities of daily living (ADL) and mobility status at admission and at discharge (evaluated with the modified Barthel Index [BI] and the Rivermead Mobility Index [RMI], respectively) (33–35), and both gain and effectiveness on the rating scales. Effectiveness reflected the proportion of potential improvement achieved during rehabilitation, which was calculated with the following formula (36):

Effectiveness = (discharge score-initial score) ÷ (maximum score-initial score) × 100.

Thus, if a patient achieved the highest score after rehabilitation, effectiveness was 100%. At discharge, CNS was not evaluated because the target of the rehabilitation was functional, not neurological, recovery.

Aphasia was evaluated with the test battery, “Esame del Linguaggio–II” (EdL-II), which has been validated in Italian and is based on age ranging from 45 to 80 years (and is based on an age range of 45–80 years), with educational status ranging from 3 to 8 years (37). The EdL-II includes several tasks that evaluate Oral Production, Oral Comprehension, Repetition, Reading Comprehension, Reading Aloud, Writing to Dictation, and Copying Words. The score on each task (0–100) represents the percentage of correct responses. All controls scored 100% in every task, so the cut-off score was 100%. All aphasic patients were also given the Token Test, with scores ranging from 0 to 36, and a cut-off score for aphasia <29 (38). In line with the Token Test and comprehension items of the EdL-II, the patients with aphasia were divided into those with comprehension deficits (CD) and those without comprehension deficits (NCD). The CD patients included those with Wernicke's and global aphasia, whereas the NCD group included patients with Broca's aphasia.

Unilateral spatial neglect (USN) was diagnosed in patients who scored below the cut-off in three of four tests of a specific standardized battery, i.e., the Letter Cancellation Test, the Barrage Test, the Sentence Reading Test and the Wundt-Jastrow Area Illusion Test (39).

In line with Bamford et al. (40), ischemic lesions were classified as total anterior circulation infarcts (TACI), partial anterior circulation infarcts (PACI), posterior circulation infarcts (POCI), and lacunar infarcts (LACI).

### Mood Evaluation

Evaluation of mood disorders was performed according to the protocol of an Italian multicenter study on PSD, i.e., DeSTRO (41), upon admission and 2 weeks later to rule out any cases of transient sad mood. In particular, patients were classified as depressed if they showed clinically evident and stable symptoms of depression during the hospital stay and obtained a Beck Depression Inventory (BDI) score of 10 or more (42, 43). The BDI has been validated in stroke patients (44), and a cut-off score ≥ 10 has been adopted in stroke patients (13). Aphasic patients were enrolled if they indicated the “sad face” on the Visual Analog Mood Scales (VAMS) (45, 46). Symptoms of depression were also classified according to the DSM-IV-TR and, more recently, the DSM-V criteria (47, 48). The only exclusion criteria for aphasic patients were a comprehension deficit when assessed with the Token test (38), with a cut-off score of 29, and no response to administration of the VAMS.

Evaluation of the patient's behavior and an interview with caregivers were conducted only by neurologists. In case of disagreement at the second evaluation, the patient was classified as non-depressed.

### Study Matching Procedures

PSD patients were then matched with patients without depression who had the same neurological impairment severity (CNS score at admission), age (difference within 1 year) and OAI (difference within 3 days), but no PSD. Patients were matched by a biomedical engineer (M.I.), regardless of whether or not they were included in the present sample, to ensure that the physicians were unaware of both the study design and the patients' outcome. The final sample included 280 patients with PSD and 280 without PSD. This study was conducted in accordance with the STROBE Statement, the Checklist of items that should be included in reports of case-control studies, added as Supplementary Material.

### Treatments

#### Physical Rehabilitation

The rehabilitation plan, which was based on practical ADL skills, was designed by physiatrists for all patients. Individual physiotherapy was performed 6 days a week for 60 min twice a day; it included physical therapy plus training for neglect or speech therapy or individual training for swallowing, bowel, and bladder dysfunctions, depending on the patient's rehabilitative needs. All rehabilitation treatment began within 24 h from admission.

Physiotherapy and language treatment continued throughout the hospital stay and the training for neglect lasted 8 consecutive weeks.

#### Pharmacological Treatment

After the second evaluation, all depressed patients received antidepressant drugs (AD), mainly citalopram and escitalopram, orally, in accordance with Italian Guidelines SPREAD (49), with progressive titration up to 20 mg per day for citalopram and 10 mg for escitalopram, but also up to 40 or 20 mg, respectively, in cases of poor response.

### Data Analysis and Statistics

Initially, we compared demographic, clinical, neuroradiological, and functional data of the two matched subgroups (according to the presence or absence of PSD) using parametric or non-parametric analyses.

Then, we compared the functional results of PSD patients based on their different response to AD treatments. Regarding depressive symptoms, we considered patients as responders if an improvement of at least 50% was observed on a depression rating scale and as non-responders if an insufficient response to treatment was observed after an adequate length of treatment and increased dosage (50, 51).

Lastly, to identify prognostic factors associated with response to antidepressant treatment, we performed a logistic regression analysis using non-response to antidepressants as the dependent variable (coded as 0 = absent and present = 1). Independent variables, all dichotomous, were: sex, age <50 years old, 50–64 years old, 65–74 years old, ≥ 75 years old, side of motor weakness, vocational status, basic schooling (≤ 8 years vs. > 8 years), malnutrition (body mass index [BMI] <18.5), PACI, TACI, LACI, POCI, presence of hypertension, diabetes, heart disease, aphasia with and without a comprehension deficit, severe disability (BI <20), severe neurological impairment (CNS score ≤ 5.5), and severe depression (BDI score > 30).

Data analyses were performed using the Statistical Package for Social Science (SPSS) 17.0. In the medical literature, an event-per-variable of 10 is widely used as the lower limit for developing prediction models that predict a binary outcome (52). This minimal sample size criterion has often been accepted as a methodological quality item in appraising published prediction modeling studies, despite some criticisms (53). To avoid possible biases, in this study the event-per-variable was increased up to 14 for the 20 analyzed independent variables, obtaining a required sample size of 280 patients.

## Results

Matching with respect to severity of neurological impairment, age, and OAI was carried out for 280 patients with PSD and 280 patients without PSD. Personal and clinical characteristics of the subgroups are shown in Table 1. The basal disability of patients with PSD was significantly more severe, both on ADL (measured with BI) and mobility (measured with RMI), than PSD- patients (than patients without PSD) (*p* = 0.035, *z* = −2.11 and *p* = 0.018, *z* = −2.36, respectively, Mann-Whitney Test). Another difference was found in the vocational status percentage, which was higher in PSD patients. No significant differences were found for the remaining parameters, such as personal data (sex, BMI, and years of schooling), comorbidities (hypertension, diabetes, heart disease), types of infarction, and neuropsychological features.

**Table 1:** Basal characteristics of the two subgroups after matching for neurological impairment, age, and onset-admission interval.

	**Non-PSD^**−**^**	**PSD**	**Sig**.
CNS score at admission	5.61 ± 2.17	5.61 ± 2.17	N.S
Age	70.44 ± 9.97	70.52 ± 10.03	N.S.
OAI	19.24 ± 11.62	19.27 ± 11.68	N.S.
Male sex	153 (54.6%)	142 (50.7%)	N.S.
Years of schooling	9.01 ± 4.80	9.39 ± 4.84	N.S.
Vocational status	19.3%	27.5%	0.022, *X*^2^ = 5.27
Right motor weakness	56.4%	51.8%	N.S.
Hypertension	61.8%	61.8%	N.S.
Heart diseases	38.2%	40.0%	N.S.
Diabetes	23.9%	23.6%	N.S.
BMI	25.07 ± 4.76	24.79 ± 4.44	N.S.
Malnutrition (BMI <18.5)	4.8%	5.2%	N.S.
PACI	50.4%	52.5%	N.S.
TACI	21.8%	26.8%	N.S.
LACI	11.8%	11.4%	N.S.
POCI	14.3%	9.3%	N.S.
Aphasia without comprehension deficit	14.6%	13.6%	N.S.
Aphasia with mild comprehension deficit	24.3%	24.3%	N.S.
Unilateral spatial neglect	21.1%	25.4%	N.S.
BDI score at admission	8.01 ± 0.98	39.67 ± 5.89	0.000 *z* = −21.12
BI score at admission	27.47 ± 25.77	22.81 ± 22.96	0.035 *z* = −2.11
RMI score at admission	2.34 ± 3.01	1.82 ± 2.58	0.018 *z* = −2.36

*N.S, non-significant; CNS, Canadian Neurological Scale; BDI, Beck Depression Inventory; BI, Barthel Index; RMI, Rivermead Mobility Index*.

As shown in Table 2, the PSD patients showed significantly greater disability at discharge on both BI and RMI (*p* = 0.007, *z* = −2.67 and *p* = 0.005, *z* = −2.83, respectively, Mann-Whitney Tests). Moreover, the effectiveness of treatment on both ADL and mobility was worse in PSD patients (*F* = 4.44, *p* = 0.036 and *F* = 6.98, *p* = 0.009, respectively), and their length of stay (LOS) was longer (*F* = 43.24, *p* < 0.001). However, at discharge, both subgroups showed a significant improvement in the BI score (*z* = −12.63, *p* < 0.001 for patients without depression and *z* = −13.68, *p* < 0.001 for PSD patients, Wilcoxon Signed Ranks Test) and the RMI score (*z* = −12.42, *p* < 0.001 for patients without depression and *z* = −13.29, *p* < 0.001 for PSD patients, Wilcoxon Signed Ranks Test). At discharge, improvement of depressive symptoms was observed in the PSD subgroup, with a significant reduction in the BDI score (*z* = −14.05, *p* < 0.001, Wilcoxon Signed Ranks Test).

**Table 2:** Rehabilitation results of the two subgroups (N.S. = non-significant).

	**Non-PSD**	**PSD**	**Sig**.
BI score at discharge	64.71 ± 30.09	57.44 ± 30.66	0.007, *z* = −2.67
RMI score at discharge	7.46 ± 4.85	6.22 ± 4.51	0.005, *z* = −2.83
Effectiveness on BI	55.47 ± 33.08	49.24 ± 31.89	0.036, *F* = 4.44
Effectiveness on RMI	42.50 ± 31.66	35.21 ± 28.86	0.009, *F* = 6.98
BDI score at discharge	7.90 ± 1.80	15.54 ± 6.42	<0.001 *z* = −19.37
LOS	64.25 ± 32.00	81.23 ± 28.94	<0.001, *F* = 43.34
Deaths	5.4%	1.4%	0.001, *X*^2^ = 11.08
Transfers	14.6%	6.1%	0.010, *X*^2^ = 6.59

*LOS, length of stay; BDI, Beck Depression Inventory; BI, Barthel Index; RMI, Rivermead Mobility Index; LOS, Length of stay*.

Finally, a higher percentage of drop-outs (i.e., due to transfer or death) was observed in patients without depression (transfer, χ^2^ = 11.08, *p* = 0.001, and χ^2^ = 6.59, *p* = 0.010 for death, respectively). However, no difference was found between subgroups regarding the three main reasons for dropping out (i.e., new strokes, pneumonia, or cardiovascular complications); however, we found a trend in patients without depression in which the percentage of both new non-fatal strokes (6 cases, 2.14% vs. 1 case, 0.36%, respectively) and deaths due to cardiovascular events (13 cases, 4.64% vs. 4 cases, 1.43%, respectively) were higher.

**Figure 1:**
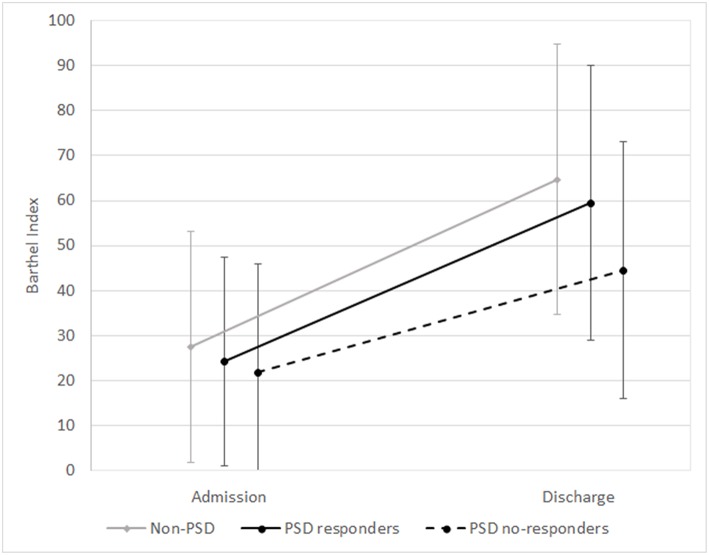
Means and standard deviations of Barthel Index scores at admission and discharge in the subgroups (non-PSD, PSD responders, and PSD non-responders). Non-PSD discharge vs. baseline *z* = −12.63, *p* < 0.001. PSD responders: discharge vs. baseline *z* = −12.77, *p* < 0.001. PSD non-responders: discharge vs. baseline *z* = −4.94, *p* < 0.001. (Wilcoxon Signed Ranks Test).

**Figure 2:**
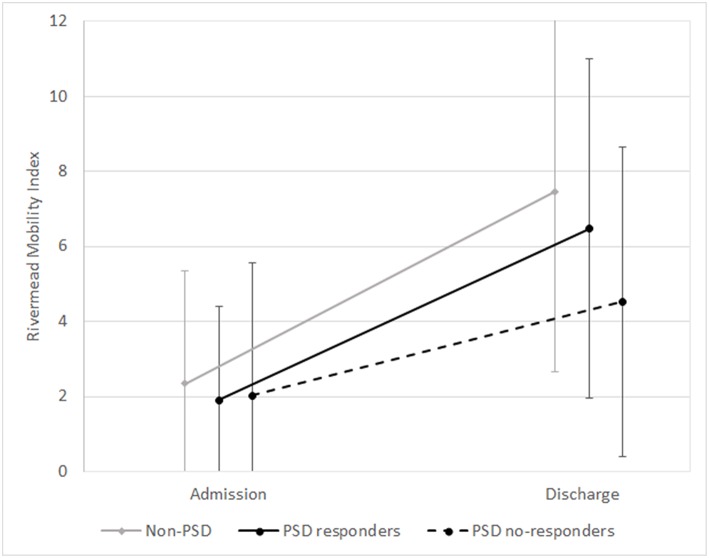
Means and standard deviations of Rivermead Mobility Index scores at admission and discharge in the subgroups (non-PSD, PSD responders, and PSD non-responders). non-PSD discharge vs. baseline *z* = −12.42, *p* < 0.001. PSD responders: discharge vs. baseline *z* = −12.37, *p* < 0.001. PSD non-responders: discharge vs. baseline *z* = −4.89, *p* < 0.001. (Wilcoxon Signed Ranks Test).

Of the 259 PSD patients, 34 patients (13.13%) who ended treatment showed an inadequate response to antidepressant therapy and were considered non-responders. As shown in Table 3 and in Figures 1, 2, PSD patients who were non-responders exhibited worse rehabilitation outcomes than patients who were responders, for both ADL and mobility, but the medical prognosis was not worse. In fact, no drop-out was observed in this subgroup. However, at discharge significant improvement compared to admission values was also observed in these patients.

**Table 3:** Rehabilitation results of PSD patients according to therapeutic response (PSD responders and PSD non-responders; N.S. = non significant).

	**PSD responders (No. 225)**	**PSD non-responders (No. 34)**	**Sig**.
BDI score at admission	39.63 ± 6.01	39.86 ± 5.34	N.S.
BDI score at discharge	13.18± 1.24	31.20 ± 4.65	0.000 *z* = −10.17
BI score at admission	24.21 ± 23.27	21.71 ± 24.14	N.S.
BI score at discharge	59.40 ± 30.56	44.50 ± 28.50	0.009 *z* = −2.61
Effectiveness on BI	51.30± 32.00	35.56 ± 27.87	0.007, *F* = 7.38
RMI score at admission	1.90 ± 2.49	2.03 ± 3.53	N.S.
RMI score at discharge	6.47± 4.52	4.53 ± 4.12	0.015 *z* = −2.42
Effectiveness on RMI	36.88 ± 29.25	24.18 ± 23.67	0.016, *F* = 5.83
LOS	82.27 ± 27.55	88.62 ± 28.63	N.S.

*LOS, length of stay; BDI, Beck Depression Inventory; BI, Barthel Index; RMI, Rivermead Mobility Index; LOS, Length of stay*.

In the logistic regression, with the occurrence of a non-response to antidepressants as the dependent variable, the presence of USN was the only prognostic factor associated with this variable (OR = 6.03, 95% CI 1.46–24.93. Significance of model: χ^2^ = 4.33, df = 1, *p* = 0.037, accuracy in prediction 90.1%). In fact, USN was observed in 14 out of 34 PSD patients who were non-responders (41.2%) vs. 21.8% in PSD patients with an adequate therapeutic response (χ^2^ = 6.04, *p* = 0.014).

## Discussion

Our data confirm that PSD is a common complication in stroke survivors and that depression is a relevant, additional disabling factor which is responsible for ~15% of the increased disability observed in patients with PSD. Our case series should be considered adequate in representing this complication, as the frequency of depression observed in our study (32.25%) is in agreement with that reported in previous studies (1). The small number of depressed patients excluded due to pre-stroke psychiatric history does not correspond with a recent meta-analysis (54), but this difference can be explained by the exclusion criteria (i.e., only patients who had been admitted to the psychiatric ward were excluded from the previous study).

Increased disability in the PSD group was mainly due to the presence of PSD, because the study design ruled out the influence of other well-known prognostic factors, such as neurological severity, age and OAI (26–30, 55). Therefore, compared to patients without depression, PSD patients showed greater basal disability on ADL and mobility, lower effectiveness of treatment and a longer LOS. However, it is important to note that disability and depression are strictly intertwined and disability *per se* may be a relevant factor for the development of depression, as shown by a recent meta-analysis (56). Moreover, just as there is a clear relationship between improved mood and improved functional recovery (14, 23), the reverse is also true, i.e., good rehabilitation results also improve mood (57).

Lastly, the medical prognosis of PSD patients was not worse than that of patients without depression, as a higher percentage of dropouts was observed among non-depressed patients. In particular, the rate of deaths does not coincide with that reported in previous studies (2–4), and the difference might be due to a slight, further antiplatelet action of treatment with a selective serotonin reuptake inhibitor (SSRIs), which is given to all depressed patients.

Therefore, PSD has an independent role in increasing disability in stroke survivors, as demonstrated in previous studies (18–22). However, in agreement with previous reports (18, 58), each group showed a significant improvement in the depression score and functional status over the course of rehabilitation.

Our study confirms the adequate efficacy of SSRIs in improving depressive symptoms. In fact, the percentage of non-responders was lower (13.13%) than that observed in patients with major depression (59); however, few studies have reported percentages of responder or non-responder PSD patients (60).

It is possible that without the AD treatment received by all PSD patients, the impact of PSD on functional outcome would have been greater. This point is confirmed by the different functional responses observed in PSD patients depending on their response to antidepressant treatment. Even though all patients improved during the rehabilitation treatment, the PSD patients who responded to AD treatment showed better functional outcome compared to the non-responders. Although the role of SSRIs in promoting functional or language recovery is debatable (61–64), our data suggest that this functional improvement was more related to the reduction of depression symptoms than to the specific action of SSRIs on disability. Thus, it is very important to identify clear prognostic factors of poor response to AD treatment. In our series, the only factor that entered the model was USN, but the exclusion of patients with a relevant history of pre-depression probably ruled out some of the potential non-responder cases (65). A recent study found that in major depressive disorder and in persistent depressive disorder, specific single nucleotide polymorphisms and haplotypes in genes related to the corticotrophin-releasing hormone (CRHBP, CRHR1) and melanocortins (POMC) predicted the non-responder status (66). Therefore, it is also important to perform these studies in PSD patients. The association between USN and non-responders to AD treatment is interesting and might be due to USN patients' inaccurate self-evaluation of their own symptoms.

### Study Limitations

Our study has some limitations. First, the retrospective nature of the study could have influenced the results and it did not allow for follow-up evaluations. The second limitation is related to the nature of the study population, i.e., since the patients were admitted to the rehabilitation ward, they had severe, not mild, paralysis. Also, patients with severe aphasia were excluded. Third, the study did not assess the PSD patients' quality of life.

### Conclusions

In conclusion, PSD must be detected, correctly evaluated, and treated. However, even if treated, PSD remains a highly unfavorable prognostic factor in stroke rehabilitation. Therefore, it is necessary to examine the potential for recovery, not only in relation to demographic and neurological data, but also in relation to the presence of depression, and, if needed, to start antidepressant treatment as soon as possible. Another relevant point is that potential non-responders to ADs should be identified early to determine additional or alternative treatments.

## Data Availability

The datasets generated for this study are available on request to the corresponding author.

## Ethics Statement

At admission, participants provided written informed consent regarding the use of their clinical and demographic data for research purposes.

## Author Contributions

SP and MI have made substantial contributions to conception and design. DD, VV, PC, AS, and GM participated in the enrollment phase and carried out clinical assessment. SP, GM, and MI participated in the manuscript draft and revision process. SP, GM, and MI participated in the study design and coordination and statistical analysis. SP drafted the manuscripts. GM, VV, PC, AS, and MI participated in the manuscript revision and gave the final approval of the version.

### Conflict of Interest Statement

The authors declare that the research was conducted in the absence of any commercial or financial relationships that could be construed as a potential conflict of interest.
